# Draft genome sequence of *Streptomyces murinus* strain SPC1 with antimicrobial potential against fungal pathogens of palms

**DOI:** 10.1128/MRA.00826-23

**Published:** 2023-11-28

**Authors:** Braham Dhillon, Seemanti Chakrabarti

**Affiliations:** 1 Department of Plant Pathology, University of Florida, Fort Lauderdale Research and Education Center, Davie, Florida, USA; University of Guelph, Guelph, Ontario, Canada

**Keywords:** *Streptomyces murinus*, pentamycin, secondary metabolite, antifungal, genome, announcement

## Abstract

*Streptomyces murinus* strain SPC1, isolated from foxtail palm seeds, exhibited antimicrobial activity against fungal pathogens of palms. The assembled genome was 8.3 Mb, with 71.96% GC content, and contained 37 secondary metabolite clusters (SMCs). A complete SMC for antifungal metabolite pentamycin (fungichromin) biosynthesis was identified in SPC1 genome.

## ANNOUNCEMENT

*Streptomyces* are filamentous, Gram-positive bacteria in the phylum Actinobacteria that are found in varied environments including soil, rhizosphere, and in diverse interactions with plants ranging from endophytes to pathogens (1). *Streptomyces* produce a diverse array of antimicrobial metabolites and account for about 80% of known microbial antibiotics produced by Actinomycetes (2).

**Fig 1:**
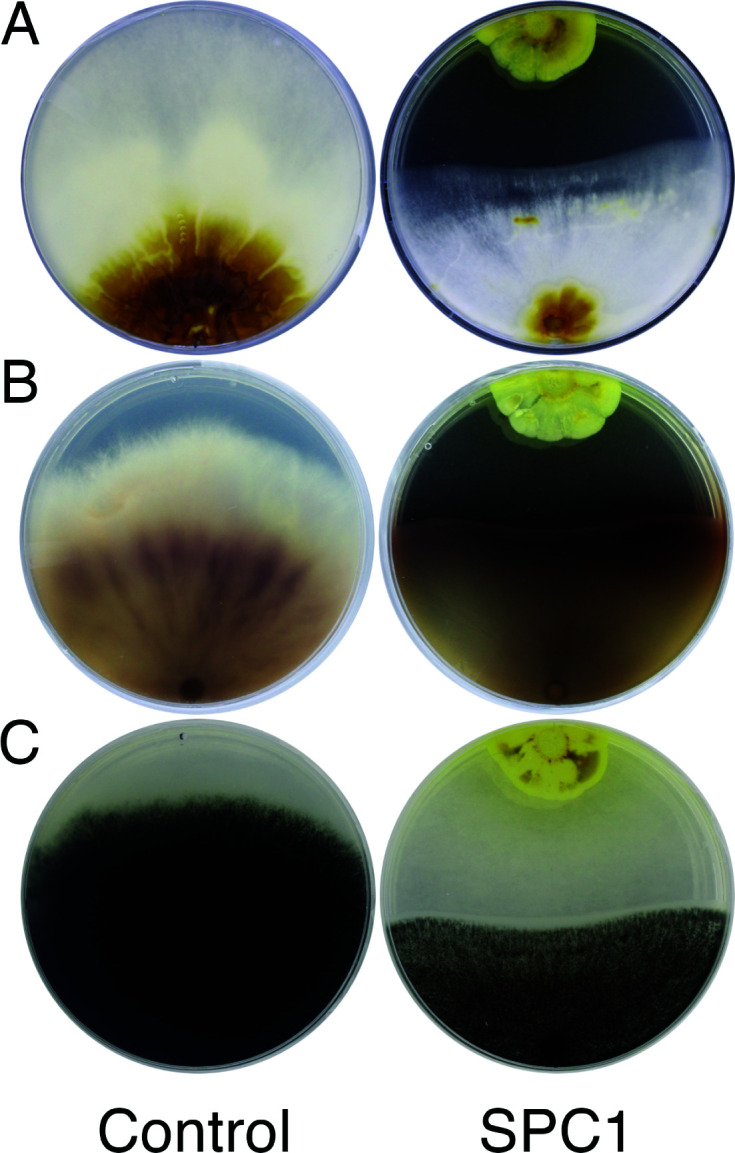
Co-cultivation assay with *Streptomyces* isolate SPC1 inhibits growth of three fungal pathogens of palms. Three palm pathogens, (**A**) *Ganoderma zonatum*, (**B**) *Fusarium oxysporum* f. sp. *palmarum*, and (C) *Thielaviopsis paradoxa*, were co-cultivated with *Streptomyces* isolate SPC1 on potato dextrose agar plates at 28°C. Inhibition of fungal growth was observed in co-culture plates as compared to the control plates.

A bacterial isolate, labeled SPC1, was recovered in Davie, FL, from ripe berries of foxtail palm (*Wodyetia bifurcata*) that had fallen to the ground upon maturity. When co-cultivated on potato dextrose agar media at 28°C, this isolate inhibited growth of three fungal pathogens of palms, *Ganoderma zonatum*, *Thielaviopsis paradoxa*, and *Fusarium oxysporum* f. sp. *palmarum* (Fig. 1). The SPC1 isolate was grown on Luria broth at 28°C for 3 d to increase biomass, and genomic DNA was extracted using Qiagen DNeasy PowerLyzer Microbial Kit. Initial sequencing of 16S rRNA gene revealed that strain SPC1 belonged to genus *Streptomyces*.

The genomic DNA was shipped to Novogene Inc. (California, USA) and sequenced on Illumina HiSeq 2000 platform (2 × 150 bp paired-end protocol). All programs were used at default settings, unless stated otherwise. The FASTQ reads were filtered (phred quality <20; length <15 bp; Ns >5) using fastp ver.0.22.0 (3, 4) and assembled with SPAdes ver.3.15.3 (5) using “--careful” flag. Completeness of genome assembly was assessed with BUSCO ver.5.3.0 (6) using streptomycetales_odb10 database. Two methods, ribosomal multilocus sequence typing (rMLST) (7) and fastANI ver.1.1 (8), were used to determine species identity. Whole-genome sequence was uploaded to PubMLST webserver (https://pubmlst.org/species-id) that identifies bacterial species using ribosome protein subunits (*rps* genes). Pairwise whole-genome average nucleotide identity (ANI) was calculated with fastANI against RefSeq genomes of 580 *Streptomyces* spp. downloaded from NCBI (accessed: 28 August 2023). Genome annotation was done using NCBI Prokaryotic Genome Annotation Pipeline (PGAP) ver.2022-04-14.build6021 (9), and secondary metabolite clusters (SMCs) were predicted using antiSMASH ver.7.0.1 webserver (relaxed; all extra features ON) (10).

Whole-genome sequencing for *Streptomyces* strain SPC1 generated a total of 17,270,456 raw reads. A data set of 17,167,820 filtered reads, equivalent to 2.6 Gb of sequence data (~310× coverage), was used for *de novo* genome assembly. The *Streptomyces* strain SPC1 assembly size was 8,311,606 bp with percent GC content of 71.96%. The assembly consisted of 25 scaffolds (N50: 445,786 bp) with the largest scaffold being 935,510 bp. Assessment of genome quality using 1,579 *Streptomycetales* ortholog groups showed 99.7% gene space completeness. The NCBI PGAP predicted 7,094 protein-coding genes, 78 tRNAs, and 3 ncRNAs in the assembled genome. The rMLST method showed 100% support for taxon identity as *Streptomyces murinus* based on exact matches to 48 *rps* genes. Similarly, fastANI computed the highest whole-genome ANI value of 97.31% to *Streptomyces murinus*.

The antiSMASH pipeline identified 37 SMCs, including 7 nonribosomal peptide synthetases (NRPS), 7 type I polyketide synthases (PKS), 7 hybrid NRPS-PKS clusters, 5 terpenes, and 11 other SMCs. One interesting SMC with potential to synthesize antifungal metabolite pentamycin (fungichromin) was identified in SPC1 genome.

## Data Availability

The sequence raw reads were deposited in the NCBI Sequence Read Archive (SRA) accession number SRR20630338 under BioProject accession number PRJNA860538.
